# Differential analysis of milk fatty acids in human, Saanen goat, Holstein cow, and Jersey cow milk at different stages of lactation

**DOI:** 10.5713/ab.24.0528

**Published:** 2025-03-31

**Authors:** Yanni Wu, Xiang Cao, YuHao Wang, Rui Gao, Kun Wang, Yuan Yuan, YongJiang Mao, Xiang Chen, ZhangPing Yang, Zhi Chen

**Affiliations:** 1 College of Animal Science and Technology, Yangzhou University, Yangzhou, China; 2 Joint International Research Laboratory of Agriculture & Agri-Product Safety, Ministry of Education, Yangzhou University, Yangzhou, China; 3 School of Nursing, Yangzhou University, Yangzhou, China; 4 Jiangsu Key Laboratory of Zoonosis, Yangzhou University, Yangzhou, China

**Keywords:** Breast Milk, Fatty Acids, Holstein Cow, Jersey Cow, Saanen Goat

## Abstract

**Objective:**

The objective of this study was to investigate the differences in fatty acid composition of milk from Holstein cows, Jersey cows, Saanen goats, and humans at different lactation period and to find possible milk that is more suitable as a base for infant milk powder.

**Methods:**

Human breast milk, Saanen milk, Holstein milk, and Jersey milk were collected at different lactation stages. Gas chromatography was used to determine the fatty acid composition of breast milk, Saanen goat’s milk, Holstein cow’s milk and Jersey cows at different stages of lactation. Pearson correlation analysis was used to analyze the correlation between different fatty acids.

**Results:**

The results show that the types and relative contents of fatty acids varied among the different varieties of milk. The main fatty acids in breast milk are palmitic acid, oleic acid, and linoleic acid, which oleic acid is the most abundant. Saturated fatty acids (SFAs) and monounsaturated fatty acids (MUFAs) differed between lactation stages, while polyunsaturated fatty acids (PUFAs) did not differ significantly. Cow’s milk and goat’s milk were mainly dominated by SFAs, and Chinese Holstein cow’s milk had the highest SFA content (72.54%). Caprylic and capric acids in SFAs were the characteristic fatty acids of goat’s milk, and their contents were significantly higher than those of breast milk and cow’s milk (p<0.05). The ratio of SFA:MUFA:PUFA breast milk was 1.72:1.45:1, Saanen goat’s milk was 14.47:6:1, Chinese Holstein cow’s milk was 14.98:4.84:1, and Jersey cow’s milk was 13.32:4.47:1.

**Conclusion:**

None of the ruminant milk has the same proportion of fatty acids as breast milk, so none of them can completely replace breast milk. From the perspective of fatty acids, it is a better choice to choose Saanen goat milk or Jersey milk as a base and then add other ingredients to form infant milk powder.

## INTRODUCTION

Humans have been herding and domesticating cattle and sheep in Africa for at least 8,000 years. Some studies indicate that humans initiated the consumption of dairy products over 6,000 years ago [1]. Most human milk is dynamic and exhibits high inter- and intra-individual variability. In recent times, we have become more concerned with the various nutrient components of dairy products, especially in the context of breastfeeding. Milk is rich in a large number of nutrients, including all kinds of biologically active substances in addition to high quality proteins, milk fat, lactose, rich minerals, and vitamins [2]. Milk can cover the full cycle of human life—the infant stage, the adolescent stage, and the old age stage. Lipids in milk, although relatively low in total milk composition, account for more than 50% of the daily intake of newborn mammals in terms of energy supply [3]. Currently, infant formulas are considered the best alternative for meeting the needs of infants who are unable to breastfeed. Cow’s and goat’s milk are often used as a base for infant formulas. However, the infant formulas currently on the market differ significantly from breast milk in terms of the structure and composition of the ingredients [4,5]. Therefore, bringing infant formulas closer to breast milk compositions has been a challenge. Among domestic animals, donkey milk is the most similar in composition relative to breast milk [6–8], followed by mare’s milk [9]. Donkey milk is high in whey protein and has the closest nutritional ratio to breast milk. The ratio of whey to casein in breast milk is 70:30, while in donkey milk, it is 64.3:35.7. Similarly, donkey milk, like breast milk, contains the enzyme lysozyme, which provides immunization for the infant. Unlike donkey milk, mare’s milk contains a higher protein content but has a lactose content that is similar to breast milk, which aids in the growth of intestinal microorganisms and calcium absorption. In addition, milk from small ruminants such as sheep, camels, and yaks represents a valuable resource because they are more productive and readily available. A comprehensive understanding of the differences between human milk and other types of milk can facilitate the design of humanized infant formulas. This includes a number of influencing factors, such as protein contents, micronutrients, vitamins, fatty acid (FA) contents, and types. All these factors affect the nutrition provided by the infant formula, as they are essential for the development and production of milk powder.

One of the most important components of milk is FAs. They serve to provide energy and increase immunity, and they act as key components in assisting the hormonal system and the metabolism of fats, carbohydrates, and proteins [10]. The structural disparities between FAs determine their biodiversity and give them special physiological importance. Fatty acids can be categorized according to their carbon chain length into short-chain fatty acids (SCFAs; 1–5 carbon atoms), medium-chain fatty acids (MCFAs; 6–12 carbon atoms), long-chain fatty acids (LCFAs; 13–21 carbon atoms), and very-long-chain fatty acids (VLCFAs; more than 21 carbon atoms) [11]. In addition, the degree of the saturation of FAs can be divided into saturated fatty acids (SFAs, without double bonds) and unsaturated fatty acids (UFAs; with double bonds); UFAs, including monounsaturated fatty acids (MUFAs; with one double bond); and polyunsaturated fatty acids (PUFAs; with two or more double bonds) [12]. Milk fat is considered to be the most complex fat in the human diet, and the quantitative composition of FAs in milk fat varies under the influence of various factors, such as animal husbandry, breed, stage of lactation, individual characteristics, climatic conditions, health, and age [13]. Studies have shown that C4–C14 and half of the C16:0 in milk FAs are produced in the mammary glands of dairy cows, and they are usually synthesized de novo under the control of sterol regulatory element-binding protein 1, acetyl-CoA carboxylase α, fatty acid synthase, and stearoyl-CoA desaturase (SCD). The remainder of LCFAs is derived from exogenous blood transformations, and they are absorbed from plasma through fatty acid-binding protein 3, acyl-CoA synthetase long-chain family member 1, and solute carrier family 27 member 6. These FAs in the mammary gland can be desaturated by SCD and then secreted into milk in the form of fat globules via diacylglycerol O-acyltransferase 1, diacylglycerol O-acyltransferase 2, and glycerol-3-phosphoacyltransferase 1. LCFAs in the blood, on the other hand, are mainly derived from feed, rumen microorganisms, or body fat mobilization [14].

Cow’s and goat’s milk have a great advantages in the application of commercial infant formulas due to the rich contents of nutrients and bioactive compounds. Overall, it is definitely not only FAs that determine the differences among various types of milk. The type and content of protein, vitamins, minerals, etc., all play an important role. Notably, there is a wide variety of FAs, which vary significantly in different types of milk and at different times. In our view, an in-depth understanding of the differences among the different lactation stages of humans, goats, and cows is essential for the development of functional dairy products and infant formula milk powder. In this study, we employed gas chromatography–tandem mass spectrometry (GC-MS) to explore the compositional changes in the FAs of human, cow, and goat milk at different lactation stages.

## MATERIALS AND METHODS

### Materials

Human milk (January 2021 to September 2022) was provided by 20 volunteers who were in their breastfeeding period in Yangzhou City, Jiangsu Province, China. The inclusion criteria were as follows: (1) healthy breastfeeding women aged 20 to 36 years who were 0 to 40 days postpartum; (2) singleton pregnancy, full-term infants, with a birth weight ≥2,500 g; and (3) those currently exclusive breastfeeding. The exclusion criteria were as follows: (1) mothers with any known or unknown infectious disease, malignant wasting disease, malnutritional disease, psychiatric disease, any pregnancy complications, and breast-related disease; (2) smokers and drinkers of alcohol, etc., and vegetarians on a vegan diet during pregnancy or breastfeeding; and (3) those currently participating in or have participated in another research project within the past 30 days. A total of 103 breast milk samples were collected, of which 16, 50, and 37 comprised colostrum (1 to 7 d), transitional milk (7 to 14 d), and mature milk (>14 d), respectively [14]. All samples were placed in breast milk storage bags, refrigerated at 4°C and brought back to the laboratory and storage at −80°C. for milk composition and FA determination. This experiment has passed the ethics review of Yang-zhou University, No: 202103085.

Goat milk: Goats with 2 to 4 parities were selected from the Saanen Goat Original Breeding Farm, Northwest Agriculture and Forestry University (Shaanxi, China). Three, three, twelve, and sixteen healthy Saanen goats in early lactation (EL) (7 to 20 d after calving), peak lactation (PL) (21 to 120 d), mid-lactation (ML) (121 to 210 d), and late lactation (LL) (211 d-300 d), respectively, were used, and the goat milk samples were selected at 50 mL/goat each; they were refrigerated at 4°Cand brought back to the laboratory and stored at −80°C for milk composition and FA determination. The feeding and management modes of dairy goats during lactation were executed exactly according to the actual feeding and management modes of goat farms.

Chinese Holstein cow’s milk: healthy 2 to 3 parity lactating Chinese Holstein cows were selected from the experimental farm of Yangzhou University. Healthy Holstein cows at the beginning of lactation (within 7 to 15 d after calving), peak of lactation (16 to 100 d), middle lactation (101 to 200 d), and LL (201 to 305 d) numbered 8, 22, 36, and 42 cows, respectively, and the milk samples were collected at 50 mL/cow and refrigerated at 4°C. They were then brought back to the laboratory and stored at −80°C for milk composition and FA determination. Lactation and Chinese Holstein cattle feeding management processes were implemented in accordance with the actual feeding management mode of the farm, and the total mixed ration (TMR) meth-od for feed was administered twice a day.

Jersey cow milk: healthy 2 to 3 parity lactating Jersey cows were selected from a farm in Sheyang County, Xuzhou City, Jiangsu Province, China, and 22, 29, 39 and 27 healthy Jersey cows were observed at the early stage of lactation (7 to 15 d after calving), at the peak stage of lactation (16 to 100 d), at the middle stage of lactation (101 to 200 d), and at the late stage of lactation (201 to 305 d), respectively. Milk samples were collected at 50 mL/cow, kept under refrigeration (4°C), brought back to the laboratory, and stored at −80°C for milk composition and FA determination. The feeding management mode of lactation and Jersey cows was in accordance with the actual feeding management mode of the farm, and the cows were fed twice a day with respect to the TMR.

The sampling of Chinese Holstein cow’s milk, Jersey cow milk, and Saanen goat milk was carried out with reference to the International Dairy Federation (IDF) standard procedure IDF 050 - ISO 707 (2008) for standard sampling and the identification of samples.

### Fatty acid extraction

In addition to the standard of extracting omega-3 and omega-6 FAs from IDF (ISO 23065|IDF 211:2009), the detection of FAs mainly refers to the Chinese National Standard (GB5009.168-2016)12, which is detected via the external standard method [15]. The specific method is as follows: Firstly, the milk samples were removed from the ultra-low temperature refrigerator, thawed at room temperature, and mixed thoroughly; then, 12 mL of milk samples was placed in 50 mL centrifuge tubes. Then, 18 mL of an n-hexane/isopropanol solution was added to the milk sample, mixed and shaken for 1 min (taking care to deflate at the right time), and centrifuged for 10 min at 1,435×g/min to remove the supernatant. Then, 8 mL of acetyl chloride methanol solution was added and mixed well, and it was placed in an incubator at 50°C for 90 min. Then, 12 mL of refrigerated 6% potassium carbonate solution was slowly added, mixed well until no bubbles are produced, and centrifuged at 176×g/min for 10 min to remove the supernatant. The sample was transferred to a 50 mL centrifuge tube. Then, 0.5 g of anhydrous sodium sulfate was added, and the sample was shaken gently to homogenize. It was then allowed to stand at −20°C for 30 min. After passing through a 0.45 μm filter membrane, it was transferred to a gas chromatography vial for online detection.

### Chromatographic conditions

Chromatographic detection conditions refer to the Chinese national standard GB 5009.168-2016. The measurement conditions of the gas chromatograph were set as follows: column, DB-23 type, 60 m×0.25 mm, 0.25 μm, Agilent, USA. The carrier gas comprised nitrogen with a flow rate of 1.0 mL/min, a split ratio of 10:1, an inlet temperature of 250°C, a pressure of 34.689 Pa, a flow rate of 14.0 mL/min, and a spacer purge flow rate of 3.0 mL/min. The detector temperature was 300°C, the flow rate of hydrogen was 30 m L/min, the flow rate of air was 350 mL/min, and the flow rate of the tail wind was 25 mL/min. The initial temperature of the column chamber’s programmed temperature increase was 130°C, and it was held for 5 min. The temperature was increased to 190°C at 6°C/min and stayed there for 20 min; then, the temperature was increased to 220°C at 2°C /min and kept there for 15 min.

### Detection data

The impurity peaks were excluded from the chromatogram, all FA methyl esters were detected, the FA methyl ester peak areas were summed, and the percentage of the individual FA methyl ester peak area over the total FA methyl ester peak area was the relative percentage of the total FA. The reference chromatogram of the standard solution of 37 FA methyl esters refers to GB5009.168-2016. The peak order of the 37 FAs was C4:0, C6:0, C8:0, C10:0, C11:0, C12:0, C13:0, C14:0, C14:1n5, C15:0, C15:1, C16:0, C16:1n7, C17:0, C17:1n7, C18:0, and C18:1n9t; and C18:1n9c, C18:2n6t, C18:2n6c, C20:0, C18:3n6, C20:1, C18:3n3, C21:0, C20:2, C22:0, C20:3n6, C22:1n9, C20:3n3, C20:4n6, C23:0, C22:2, C24:0, C20:5n3, C24:1n9, and C22:6n3. SFAs include C4:0, C6:0, C8:0, C10:0, C11:0, C12:0, C13:0, C14:0, C15:0, C16:0, C17:0, C18:0, C20:0, C21:0, C22:0, C23:0, and C24:0. PUFAs include C18 :2n6t, C18:2n6c, C18:3n6, C18:3n3, C20:2, C20:3n6, C20:3n3, C20:4n6, C22:2n6, C20:5n3, and C22:6n3. MUFAs include C14:1n5, C15:1, C16:1, C17:1, C18 :1n9t, C18:1n9c, C20:1, C22:1n9, and C24:1. Ω-3 PUFA include C18:3n3, C20:3n3, C20:5n3, and C22:6n3. Ω-6 PUFAs include C18:2n6t, C18:2n6c, C18:3n6, C20:3n6, and C20:4n6. Linoleic acid (LA) includes C18:2n6t and C18:2n6c (The standard 37 FA methyl ester chromatograms are shown in Supplements 1,2, and the sample FA chromatograms are shown in Supplement 3).

### Data processing and statistical analysis

The relevant data were preliminarily organized in Excel and statistically analyzed using SPSS 27.0 software; the indicators were subjected to one-way ANOVA and LSD (least significant difference) tests and plotted using Origin 9.1.

## RESULTS

### Analysis of fatty acid compositions in different lactation phases of breast milk

In this experiment, we measured a total of 31 breast milk FAs (Supplement 4), encompassing 14 SFAs, 7 MUFAs, and 10 PUFAs. As shown in Table 1, the three most abundant FAs in breast milk are oleic acid (C18:1n9c), palmitic acid (C16:0), and LA (C18:2n6), which account for 31.1%, 24.5%, and 20.4% of the total FAs, respectively. In different lactation periods, the relative content of breast milk FAs also produced changes, and we found that as lactation progressed, the relative content of SFA 8:0 to C15:0 gradually increased (p<0.05). Moreover, the relative contents of C20:3n3, C20:4n6, C24:0, C20:5n3, and C22:6n3 gradually decreased. The relative content of SFA in the transitional milk stage was about 39.1%, which was significantly lower than that of colostrum and mature milk (p<0.05). Regarding MUFAs, the opposite trend was observed, and the relative content of MUFA in transitional milk was about 34.5%, which was significantly higher than that of colostrum (p<0.05). PUFAs did not change significantly across lactation periods (p>0.05). However, we subdivided the PUFAs (into: n3-UFA, n6-UFA, and n9-UFA) and found that the relative content of n3-UFA was significantly lower in mature milk compared to colostrum and transitional milk; the relative content of n9-UFA was significantly higher than that of colostrum and transition milk in transition milk (p<0.05); and there was no significant change in n6-UFA. In terms of carbon chain length, breast milk FAs are mainly LCFA, which account for about 92%, and MCFA, which account for about 2%. The levels of SCFAs in the samples we tested were extremely low, almost negligible. The relative content of MCFAs was significantly higher in the mature milk stage than in colostrum and transitional milk, and the relative content of VLCFA was significantly higher in the colostrum stage than in the mature milk and transitional milk (p<0.05).

**Figure 1:**
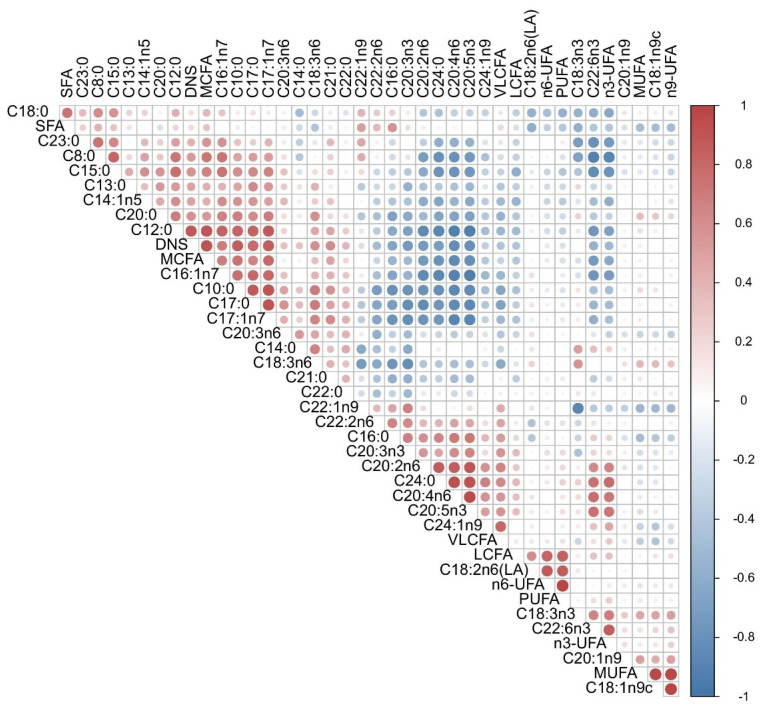
Correlations between the FAs of breast milk. Red, higher correlation; blue, lower correlation. SFA, saturated fatty acid; DNS, *de novo* synthesis fatty acid; MCFA, medium-chain fatty acid; VLCFA, very long-chain fatty acid; LCFA, long-chain fatty acid; UFA, unsaturated fatty acid; PUFA, polyunsaturated fatty acid; MUFA, monounsaturated fatty acid; FA, fatty acid.

We also analyzed Pearson correlations between FAs in milk. As shown in Figure 1, in general, the different FA compositions indicated significant correlations with each MCFA composition (C8:0–C15:0), with a cluster-like distribution. Long-chain C16:1n7 and C17:1n7 were significantly correlated with SFA C8:0–C17:0. MUFAs were positively correlated with DNSs, MCFAs, and LCFAs and negatively correlated with SFAs and VLCFAs, with a highly significant negative correlation with SFAs. In addition, we detected a negative correlation between n3-UFA and MCFA and DNS. There was no significant correlation between the n3-UFA, n6-UFA, and n9-UFA PUFAs, but n3-UFA was positively correlated with C20:4n6. Moreover, we also found that the functional C20:5n3 and C22:6n3 FAs were highly significantly positively correlated, with C20:5n3 being highly significantly negatively correlated with all MCFAs (except C14:0) and C22:6n3 also being highly significantly negatively correlated with MCFAs.

### Analysis of the milk fatty acid composition of Saanen dairy goats at different stages of lactation

In this experiment, we detected 34 FAs in Saanen milk goat milk (Supplement 5). Among them, there were 16 SFAs, 7 MUFAs, and 11 PUFAs. As shown in Table 2, goat milk FAs were dominated by C16:0 and C18:1n9c, which each accounted for 24.4% and 25% of the total FAs. Compared to breast milk, Saanen goat milk had significantly higher SFA contents at about 66.8%; on the contrary, the content of UFAs was significantly lower, and the content of PUFA was only 4.6%. The FA content of goat milk varied with the lactation period. SCFAs were significantly higher during ML than during the peak of lactation, and MCFAs were not significantly different except for C6:0. There was no significant change in the total LCFA content, but the levels of C15:0, C17:1, C18:2n6, and C20:1 were higher during the PL compared to the other lactation periods, and the level of C18:3n6 was higher during the EL compared to the other lactation periods. The relative content of SFAs in ML was about 71.8%, which was significantly higher than that in LL (p<0.05). For MUFAs, the opposite trend was observed, with the relative content of MUFAs during LL being about 29.2%, which was significantly higher than that during ML (p<0.05). Similarly, PUFAs were highest in ML at about 5.5%, which was significantly higher than in PL and LL. PUFAs are subdivided, and they are divided into n3-UFA, n6-UFA, and n9-UFA. We found that the relative content of n3-UFA was significantly higher in the EL than in other lactation periods. For n6-UFA, its relative content in ML was significantly higher than that in PL and LL, while the difference between EL and PL was not substantial. Conversely, n9-UFA was the lowest in ML.

**Figure 2:**
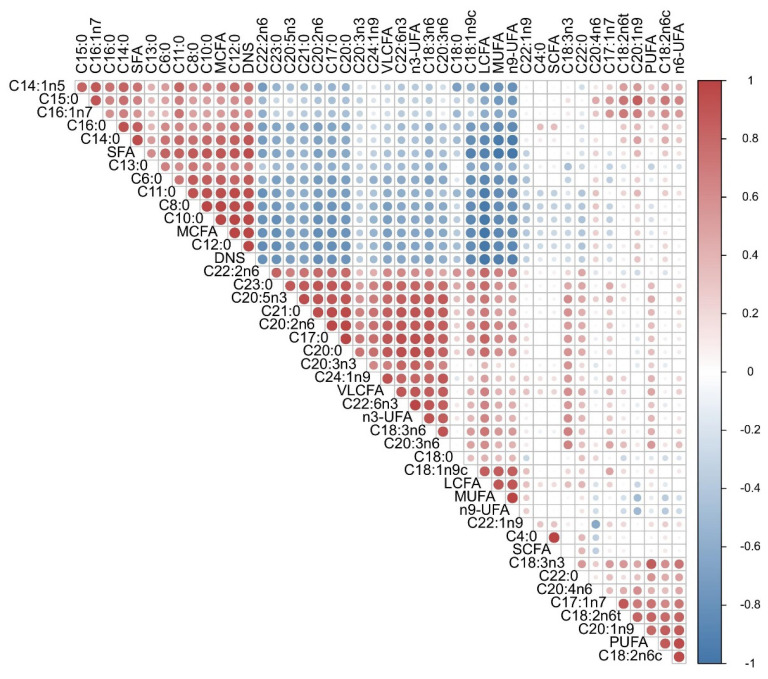
Correlations between the FAs of goat milk. Red, higher correlation; blue, lower correlation. SFA, saturated fatty acid; MCFA, medium-chain fatty acid; DNS, *de novo* synthesis fatty acid; VLCFA, very long-chain fatty acid; UFA, unsaturated fatty acid; LCFA, long-chain fatty acid; MUFA, monounsaturated fatty acid; PUFA, polyunsaturated fatty acid; SCFA, short-chain fatty acid; FA, fatty acid.

As shown in Figure 2, the Pearson’s correlation analysis of FAs in goat milk showed that, overall, the different FA components were significanly correlations with one another and a cluster-like distribution, with each MCFA component (C6:0 to C12:0) showing a significant positive correlation (p<0.05) with each other and a negative correlation with the LCFAs. The DNS components (C6:0 to C15:0) were significantly negatively correlated with LCFAs and VLCFAs and negatively correlated with n3-UFA and n9-UFA in MUFA, but not with n6-UFA. MUFA was negatively correlated with MCFAs, DNSs, and SFAs and positively correlated with VLCFAs. In addition, we detected a weak correlation between n3-UFA and n6-UFA, but n3-UFA was significantly positively correlated with C18:3n6, C20:3n6, and C20:2n6. SCFAs were not strongly correlated with the other FAs (p>0.05).

### Analysis of the milk fatty acid composition of Chinese Holstein cows at different lactation stages

**Figure 3:**
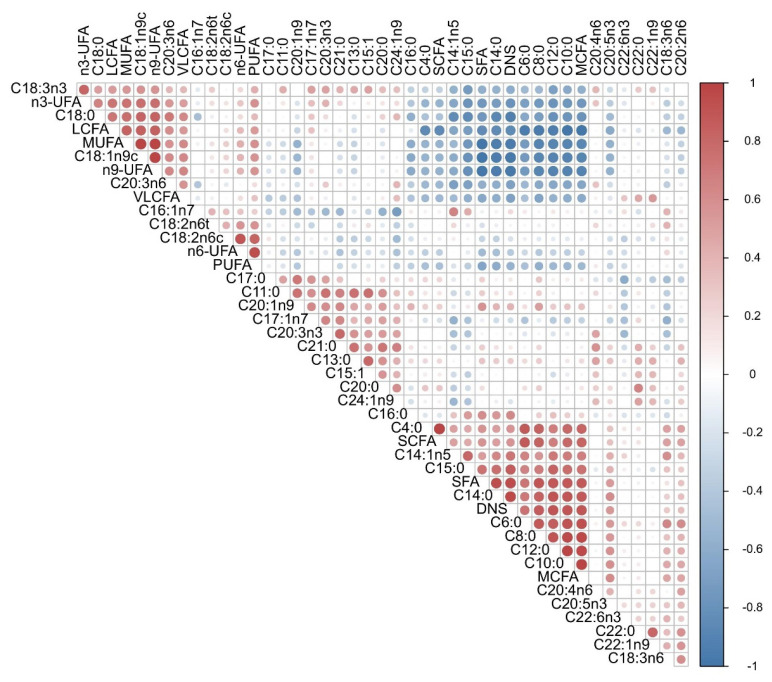
Correlations between FAs of Chinese Holstein milk. Red, higher correlation; blue lower correlation. UFA, unsaturated fatty acid; LCFA, long-chain fatty acid; MUFA, monounsaturated fatty acid; VLCFA, very long-chain fatty acid; PUFA, polyunsaturated fatty acid; SCFA, short-chain fatty acid; SFA, saturated fatty acid; DNS, de novo synthesis fatty acid; MCFA, medium-chain fatty acid; FA, fatty acid.

In the milk of Chinese Holstein cows, we detected a total of 33 FAs (Supplement 6), of which 15 were SFAs, 8 were MUFAs, and 10 were PUFAs. As shown in Table 3, the three most abundant FAs were C16:0, C18:1n9c, and C18:0 (in this order), accounting for 34.1%, 20.5%, and 11.6% of the total FAs, respectively. The relative content of SCFAs was affected by the time of lactation, with the relative content of C4:0 being significantly higher at the beginning of lactation than at other lactation periods. The relative content of MCFAs was contrary to that of SCFAs, and C6:0 did not change significantly from lactation to lactation. C8:0 showed an opposite trend to C4:0, and the relative content of C8:0 in the LL was significantly higher than that in the other lactation periods (p<0.05). The relative contents of C10:0 and C12:0 were significantly lower in the EL than in the other lactation, and the relative contents were stable from the PL to the LL, with no significant differences. The relative contents of LCFA, C15:1, C18:2n6c, C18:3n3, C18:3n6, C20:0, C20:4n6, and C21:0 were not significantly different in all four lactation periods. The relative amounts of C16:1n7, C17:0, and C17:1n7 were significantly higher in LL than in ML (p<0.05). Among the VLCFA, C22:6n3 was not detected in most samples from peak and late lactation periods, C24:1n9 was not detected in PL, and the relative contents of the remaining VLCFAs did not differ significantly between several lactation periods. For functional FAs, n3-UFA did not change significantly over several lactation periods, and the relative content of n-6 UFAs was significantly higher in LL than in EL and PL, and it was about the same as in ML. The highest relative amount of n9-UFA was found at the beginning of lactation, which may be due to the influence received from C18:1n9c, for which its amount was significantly higher at the beginning of lactation than at other lactation periods. In the present experiment, we found that SFAs increased with the prolongation of lactation and then decreased at the end of lactation, whereas MUFAs were higher in EL and LL and lower at PL, which may mainly originate from the mobilization of body fat during pre-lactation. In addition to the comparisons of relative FA contents across time, we analyzed the Pearson correlation of milk FAs in Chinese Holstein cows. As observed in Figure 3, most FAs are correlated with each other and are distributed in clusters. There is a significant positive correlation between MCFAs and LCFAs (C6:0-C15:0) and a negative correlation with C18:n UFAs. The correlation between LCFAs was not strong and there was no obvious pattern. There was a significant negative correlation between SFAs and UFAs; a positive correlation was observed between MUFA and PUFAs. For functional FAs, n3-UFA was positively correlated with n6-UFA, but the correlation was not strong, probably because of its negative correlation with C18:3n6 and C20:2n6. n6-UFA was negatively correlated with other FAs except for C18:0, C18:1n9c, C20:3n6, C16:1n7, C18:2n6, and C18:3n6, which were positively correlated, but none of the correlations were strong.

### Analysis of the fatty acid composition of milk from Jersey cows at different stages of lactation

In Jersey cattle, we detected a total of 36 FAs (Supplement 7), of which there were 17 SFAs, 8 MUFAs, and 11 PUFAs. As shown in Table 4, the three most abundant FAs were C16:0, C18:1n9c, and C18:0 in that order, accounting for 35.8%, 18.5%, and 10.6% of the total FAs, respectively. The relative content of SCFAs decreased with the prolongation of lactation, and the content of SCFAs was significantly higher in EL than in other lactation periods. The relative content of MCFAs, on the other hand, increased with the prolongation of lactation, and the relative content of MCFAs was higher at the PL and LL than at the EL and ML. The relative contents of some of the LCFAs were the same as those of MCFAs in different lactation periods, but the relative contents of four FAs—C13:0, C15:1n5, C16:1n7, and C18:3n6—did not differ significantly among several lactations, and C18:0, C18:1n9c, C18:2n6t, C18:3n3, and C20:1 and C17:0, C20:2, C20:3n6, C20:4n6, C20:5n3, and C21:0 were higher in EL and ML; they then decreased with prolonged lactation. Peculiarly, the relative amount of C17:1n7 was lowest at the PL, whereas the relative amount of C18:2n6c was higher at ML and at the PL, and the relative amount of C20:0 was highest at the beginning of lactation. The relative amounts of VLCFAs were lower and varied more regularly in several lactation periods, with most VLCFAs being higher in EL and ML, and they decreased with respect to the duration of lactation, with the exception of C22:1n9 and C22:2n6. The relative amounts of C22:1n9 did not differ significantly across the four lactation periods. The relative content of C22:2n6 increased with the duration of the lactation period. For functional FAs, n3-UFA and n9-UFA were the highest in colostrum and gradually decreased with prolonged lactation, whereas n3-UFA was highest in ML, with little difference between the other three lactation periods.

**Figure 4:**
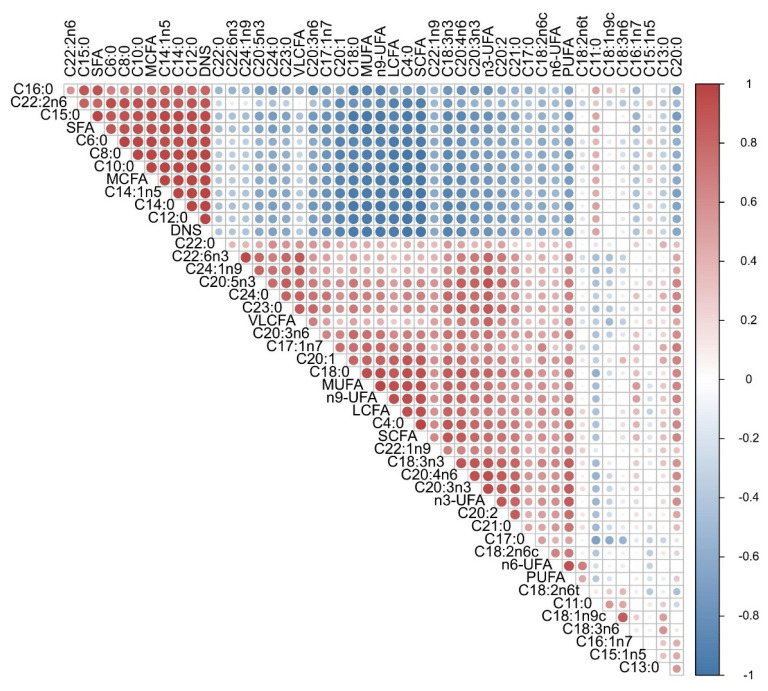
Correlations between the FAs of Jersey milk. Red, higher correlation; blue, lower correlation. SFA, saturated fatty acid; MCFA, medium-chain fatty acid; DNS, *de novo* synthesis fatty acid; VLCFA, very long-chain fatty acid; MUFA, monounsaturated fatty acid; LCFA, long-chain fatty acid; SCFA, short-chain fatty acid; UFA, unsaturated fatty acid; PUFA, polyunsaturated fatty acid; FA, fatty acid.

In addition to comparing the relative FA content across time periods, we conducted an analysis of the Pearson correlation of FAs in Jersey cow’s milk. As observed in Figure 4, the 37 FAs detected in the milk showed a significant correlation with each other and a cluster-like distribution. All MCFA components (C6:0 to C12:0) showed highly significant positive correlations with each other, but they were significantly negatively correlated with the SCFA C4:0 and negatively correlated with the LCFAs except for C14:0, C14:1n5, C15:0, and C16:0; moreover, the VLCFAs, except for C22:2n6 SFAs, were highly significantly positively correlated with MCFAs and negatively correlated with UFAs, excluding C18:2n6t and C15:1n5. Surprisingly, SFAs were negatively correlated with C20:0, C18:0, C4:0, C22:0, C23:0, C24:0, C21:0, C17:0, and C13:0. MUFAs were significantly negatively correlated with SCFAs and MCFAs (C6:0 to C16:0) but positively correlated with the SFAs C20:0, C18:0, C4:0, C22:0, C23:0, C24:0, C21:0, and C17:0. As with MUFAs, PUFAs were significantly negatively correlated with SCFAs and MCFAs (C6:0 to C16:0) and positively correlated with several SFAs, such as C18:0, C20:0, C4:0, C22:0, C24:0, C23:0, and C21:0. The analysis of functional FAs revealed that n3-UFA and n6-UFA and n9-UFA were positively correlated with each other.

### Analysis of the composition of fatty acids in different types of milk

Table 5 distinctly presents the differences in fatty acid content and types between the four types of milk. From the FA composition of the examined breast milk, goat’s milk, Chinese Holstein cow’s milk, and Jersey cow’s milk, it becomes evident that breast milk has very low levels of C4:0 and C6:0 that are approximately equal to nondetectable levels compared to goat’s milk and cow’s milk. Goat’s milk and cow’s milk contain relative levels in the range of 1% to 2%, among the three milk substitutes, Jersey cow’s milk registers the lowest levels, without any significant difference. C11:0 was also not detected in breast milk, and the relative levels in the other three milks were as meager as less than 1%, with the lowest levels in Jersey cow’s milk. Among the short- and medium-chain saturated fatty acids, C8:0 and C10:0 are the characteristic FAs of goat milk [15], and the content in goat milk is significantly higher than that of other types of milk. Furthermore, C8:0 and C10:0 have been shown to have antiviral effects [16,17], which can help improve the immunity of infants and young children. For long-chain saturated fatty acids (C13:0 to C21:0), the relative levels in breast milk were all significantly lower than those in goat and cow milk, while the relative levels in Jersey cow milk were lower than those in Saanen goat milk and Chinese Holstein cow milk. Very-long-chain saturated fatty acids (C22:0, C23:0, and C24:0) were present at very low levels (<1%) in several of the milk tested. The relative levels of C22:0 in breast milk and goat milk were significantly lower than those in Holstein and Jersey cow’s milk. C23:0 was not detected in Holstein cow’s milk, and it was present at very low levels in goat’s milk; C24:0 was not detected in goat’s milk or in Holstein cow’s milk.

For UFAs, C18:1 was the most abundant MUFA in milk, and the content of C18:1 in breast milk was significantly higher than that of the other three ruminant milks; among the three ruminant milks, the levels in the milk of dairy goats were significantly higher than that of Holstein and Jersey cows, whereas there was no significant difference between Holstein and Jersey cow milk. For PUFAs, it can be observed that breast milk has significantly higher PUFAs than ruminant milk, mainly because of the very high content of C18:2n6c, which is one of the characteristics of breast milk that gives it its nutritional value. In addition to this, n3-UFA in breast milk was significantly higher than in several other milks, and among the three ruminant species, the milk of Jersey cows had significantly higher UFA contents than that of goat and Holstein cows’ milk, which had higher nutritional value. Overall, unlike ruminant milk fat, human milk has lower levels of SFAs, along with higher MUFA and PUFAs, consistent with previous studies. Low SFA levels have been reported to be associated with a reduced risk of heart disease and to be decisive in the development of atherosclerosis [18], with goat milk having a clear advantage of low SFAs compared with cow milk.

## DISCUSSION

Breast milk provides the nutrients needed for infant growth and development. Fat, which accounts for about 3% to 5% of breast milk, is the main energy-supplying component of breast milk, providing about 50% of the energy, essential FAs, and fat-soluble vitamins for infant growth [19,20]. In recent years, extensive research has been carried out on the FA composition of breast milk, which is influenced by factors such as geographic location, dietary habits, the level of economic development, the duration of breastfeeding, and maternal genetics [21,22]. Previous studies have shown that SFAs account for about 37% to 56% of the total FAs in breast milk, MUFAs account for about 25% to 43%, and PUFAs account for about 10% to 20%, which is consistent with our findings [23,24]. In breast milk, SCFAs were not detected, while ruminants such as cattle and goats had more SCFAs in milk fat due to the fermentation of microorganisms in the rumen [25]. SCFAs can regulate lipid metabolism, reduce the risk of obesity, and protect and promote intestinal maturation and other functions [26]. The relative content of SCFAs in dairy cows and goats is significantly higher than in breast milk, which is an advantage as a basis for infant formulas. In the present study, it was found that SFAs in breast milk FAs had the highest percentage of total FAs and remained relatively stable throughout the lactation phase. Among the FAs in breast milk, SFAs are important energy-supplying components. SCFAs were not detected in most samples in this experiment, and previous studies have shown that breast milk has a very low content of SCFAs, which is only about 0.16% [4,27]. Therefore, as the lactation period extends and the infant’s energy needs increase, the amount of MCSFAs in breast milk, which play a role in energy supply, increases. It has been found that the secretion of MCFAs is triggered by childbirth, the level of MCFAs in breast milk is significantly higher after childbirth, and the biosynthesis of MCFAs predominates in human mammary epithelial cells [28,29]. This may account for the increase in MCFA content with prolonged lactation. As with breast milk, there was a similar trend towards changes in the relative levels of MCSFA (C8:0 to C15:0) in goat’s and cow’s milk, increasing after EL. This may be due to the increased energy requirement of the pups as the feeding time is extended. MCFAs delivered directly to the liver through the portal vein, which can quickly provide energy to the youngsters. In addition, MCFAs in the breast are synthesized de novo from mammary cells, and the metabolism of the mammary gland is not yet mature in the early stages of lactation. In dairy cows, changes in milk fat content are also related to actual production. To promote rapid mammary cell repair, there is usually a dry period of 2 months before calving, and appropriate supplemental feeding should be given close to the time of delivery [30]. The milk yield of cows increased gradually in the pre-lactation period, and the milk fat percentage was also at a high level. However, the milk yield increased rapidly in the PL, and the milk fat percentage decreased significantly. In the LL, the milk yield decreased gradually, while the milk fat percentage increased slowly, and the composition of FAs and their proportions in the milk changed along with the changes in the milk fat percentage [31,32].

Holstein milk is a common ingredient in infant formula due to its high yield and low cost. In contrast, Jersey milk is more costly and is produced less, but it has a higher nutritional value. Furthermore, they have lower levels of MCFAs than goat’s milk, which is more similar to breast milk. MCFAs exhibit antibacterial and antiviral effects, and they also affect the establishment of intestinal flora in infants [33]. However, an excess of MCFAs can block the absorption of other essential fatty acids in the infant and have potential toxicity relative to certain organs [34]. Previous studies have shown that breast milk contains significantly higher levels of LAs compared to other ruminant milk [35]. In addition to this, the LA content of breast milk detected in this experiment was higher than that of some previous studies. This may be due to the fact that about 30% of the LA in breast milk stems comes from the diet of lactating mothers, which is greatly influenced by the mothers’ diets. Given that Chinese residents frequently consume vegetable oils rich in LA content in their diets, such as soybean oil, corn oil, sunflower oil, and peanut oil [36,37].

Breast milk is the best source of nutrition for infants, and the content of branched-chain FAs in breast milk is as high as 1.5%, which has anti-inflammatory and anti-tumor effects, and maintains the energy balance and other beneficial effects on the human body [38]. However, affected by a variety of factors, such as lifestyle, social pressure, and individual physical conditions, the Chinese breastfeeding rate is lower than that of other countries. The demand for breast milk substitutes on the market is expanding. Goat’s milk is rich in nutrients and contains a variety of nutrients needed by the human body. Compared with cow’s milk, goat’s milk has larger fat globules (3.19 to 3.50 μm), which have a larger contact area with lipase in the gastrointestinal tract and are more easily absorbed by the body. In particular, goat’s milk has a lower casein content and is more similar in composition to breast milk, which reduces the occurrence of goat milk allergies [39]. These characteristics endow goat milk with a dominant position in the dairy industry, enabling it to develop rapidly and expand its market scale. In our tests, we found that the SFA content of Saanen dairy goat milk is significantly lower than that of cow’s milk, and the MUFA content is significantly higher than that of cow’s milk, which has higher nutritional value, and it can be used as the basic material for the research and development of formulas that are more in line with the nutritional needs of infants and young children. At present, there are more goat milk-based milk powders on the market. Compared with cow milk-based milk powder, goat milk-based milk powder has a lower moisture mass fraction and better physical and chemical properties. In previous studies, there were mixed opinions about the effects of different substrate milk powders on infant and young child development, some studies believed that goat milk powder was beneficial to infant development [40], and some studies showed that there was no difference in the development of infants fed with different milk powders [41].

Not only is the fat content much higher than that of Holstein cow’s milk but the protein content of Jersey cow’s milk is about 20% higher than that of Holstein cows [42,43]. FA composition and ratios are important indicators and evaluators of dairy quality and nutritional value. Significant differences in the FA composition among the three types of milk FAs were observed. Jersey cow’s milk contained fewer SCFAs than Holstein cow’s and goat’s milk, while most LCFAs were significantly higher than cow’s milk, and goat’s milk and Jersey cow milk fat had higher MUFAs and PUFAs and lower SFAs. The main factor affecting the percentage of each type of FA is feeding, but it is also influenced by individual differences, genetic parameters, breed, litter size, milk, fat production, stage of lactation, or metabolic status of the animal [44].

Depending on the position of the double bond, PUFAs can be divided into two types: ω-3 and ω-6. Omega-3 PUFAs (n-3 PUFA) are essential nutrients that play an important role in the human body. In recent years, with the deepening of milk research, the nutrition of milk has been gradually recognized, and the consumption of milk has increased year by year. Therefore, the development of n-3 PUFA-rich dairy products can improve people’s intake of n-3 PUFA, which is beneficial to people’s health. The n-3 PUFA in breast milk was significantly higher than that in ruminant milk, and among ruminants, the relative content of Jersey cow’s milk was again significantly higher than that of Holstein cow and Saanen dairy goat. The two important members of n6-UFA are LA and arachidonic acid (AA), which are essential FAs that can reduce blood cholesterol levels [45]. n3-UFA has been found to have a mitigating effect on cardiovascular disease and rheumatoid arthritis in the last decade or so, especially C20:5n3 (EPA) and C22:6n3 (DHA). LA and ALA are precursors for the synthesis of AA and DHA, which play an important role in the development of infants’ intelligence and vision. At the same time, DHA can enter the maturation of the baby’s retina and the development of the visual cortex. Therefore, the abundance of DHA can improve visual acuity and promote the development of the baby’s brain. LA and ALA are precursors for the synthesis of AA and DHA, which play an important role in the development of infants’ intelligence and vision [46]. These key FAs are less plentiful in cow’s and goat’s milk, which is where cow and goat milk-based infant formulas fall short. In order to be more similar to breast milk, these FAs need to be added artificially. From this point of view, compared to other types of milk, Jersey milk is more suitable as a base for milk powder because it is richer in n-3 PUFA and n-6 PUFA, which can save additional FAs. The concentration of added FAs is very important, and our study may also provide a theoretical basis for exogenous fatty acid addition. There are many studies that have tested for FAs in milk, and the results of each study vary due to the limitations of the sample size and measurement technology. In our study, we analyzed the types and relative contents of FAs in breast milk, Saanen dairy goat milk, Holstein cow milk, and Jersey cow milk via gas chromatography, and more FAs were detected in ruminant milk than in breast milk, but the relative contents varied. A greater variety of FAs were detected in ruminant milk than in breast milk, but the relative abundance varied. In terms of the content and proportion of fatty acids, there is no ruminant milk that can completely replace breast milk, but infant formula can be further improved according to the proportion of various FAs detected, which provides a certain basis for the production of infant formula. In order to comprehensively explore which milk is more suitable as a base for infant milk powder, we will also conduct a comprehensive evaluation from the perspectives of milk protein, lactose and vitamins to provide a reference for better formula design.

## CONCLUSION

**Figure 5:**
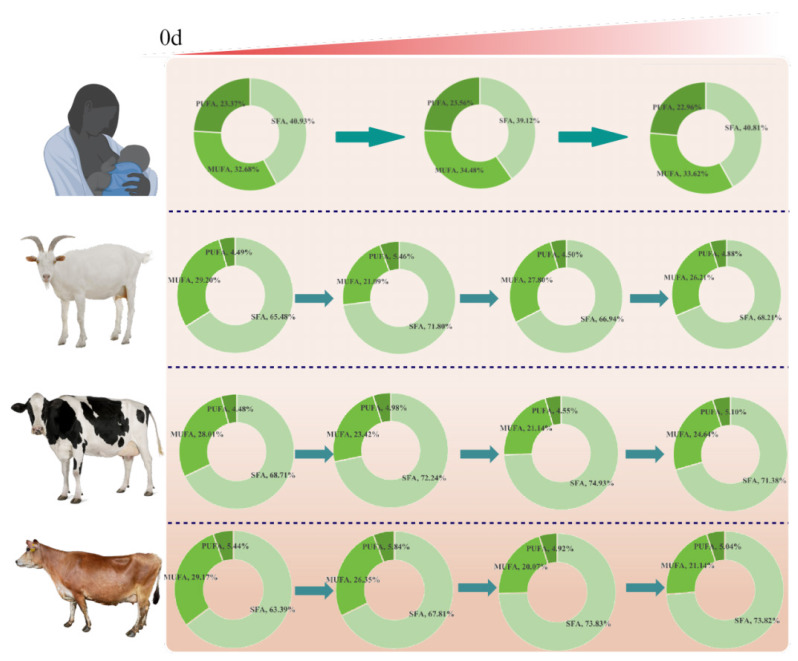
The composition of milk fatty acids in different types was different in different periods. SFA, saturated fatty acid; MUFA, monounsaturated fatty acid; PUFA, polyunsaturated fatty acid.

Each type of milk has the same type of fatty acids at different stages of lactation, but the amount is different. Compared to ruminant milk fat, breast milk has lower levels of saturated fatty acids and higher levels of MUFAs and PUFAs. Among several ruminant milks, Saanen dairy goat milk has a lower SFA content and a higher MUFA content. On the other hand, Holstein and Jersey cow’s milk have low levels of MCFAs. Jersey cow’s milk has higher levels of n3-UFA and PUFA, which are more nutritious (Figure 5). However, no single type of ruminant milk can completely replace breast milk, and the content and proportion of fatty acids in different milk can only provide a reference point for the development of breast-milk substitutes and formulas. If ruminant milk is used as the substrate, different levels of various FAs need to be added depending on the FA profile of the human milk.

## Figures and Tables

**Table 1:** Some fatty acid contents of breast milk at different lactation periods (% of total fatty acid, mean±SD)

Fatty acid	Colostrum stage	Transitional milk stage	Mature milk stage
C8:0	0.115±0.012^b^	0.131±0.011^a^	0.206±0.041^a^^b^
C10:0	0.677±0.385^b^	1.261±0.261^a^	1.249±0.233^a^^b^
C12:0	1.431±0.256^b^	3.140±0.586^a^	3.640±0.869^a^^b^
C15:0	0.137±0.028^b^	0.172±0.038^a^	0.200±0.028^a^^b^
C16:0	27.426±1.637^b^	23.755±1.475^a^	24.104±2.237^a^
C18:0	5.366±0.641	5.337±0.906	5.938±0.857^a^^b^
C18:1n9c	30.098±1.827^b^	31.664±1.915^a^	30.768±1.548^b^
C18:2n6 (LA)	19.452±5.176	20.746±2.175	20.312±2.378
DNS	7.296±0.754^b^	9.292±1.611^a^	9.726±1.636^a^
MCFA	2.061±0.570^b^	3.774±1.511^a^	4.749±1.238^a^^b^
LCFA	92.841±5.976	92.021±4.119	91.088±2.097
VLCFA	2.075±1.052^b^	1.366±0.487^a^	1.564±0.484^a^
SFA	40.927±1.871^b^	39.124±2.578^a^	40.813±2.470^b^
MUFA	32.681±1.218^b^	34.478±1.953^a^	33.624±1.625^b^
n3-UFA	2.802±0.683	2.760±0.489	1.854±0.477^a^^b^
n6-UFA	20.419±5.103	20.602±4.542	20.958±2.356
n9-UFA	31.183±1.381^b^	32.443±1.971^a^	31.474±1.539^b^
PUFA	23.368±4.907	23.560±4.652	22.964±2.491

a p<0.05 compared with the colostrum stage;

b p<0.05 compared with the transitional milk stage.

SD, standard deviation; DNS, *de novo* synthesis fatty acid; MCFA, medium-chain fatty acid; LCFA, long-chain fatty acid; VLCFA, very long-chain fatty acid; SFA, saturated fatty acid; MUFA, monounsaturated fatty acid; UFA, unsaturated fatty acid; PUFA, polyunsaturated fatty acid.

**Table 2:** Some fatty acid contents of goat milk at different lactation periods (% of total fatty acid, mean±SD)

Fatty acid	Early lactation	Mid-lactation	Peak lactation	Late lactation
C4:0	1.704±0.046	2.232±0.422^c^	1.680±0.339^b^	1.798±0.385
C6:0	2.013±0.251^b^	2.759±0.812^a^^c^	2.215±0.278^b^	2.046±0.323^b^
C8:0	2.496±0.621	2.672±0.750	2.751±0.473	2.518±0.557
C10:0	7.660±2.106	8.395±2.547	9.085±1.622	8.377±2.130
C12:0	4.158±1.731	4.073±1.666	4.107±0.808	3.886±1.094
C14:0	9.280±0.861	9.826±2.424	8.503±0.431	8.273±1.153^b^
C15:0	1.065±0.108^b^	1.482±0.509^a^^c^	1.073±0.082^b^	1.037±0.134^b^
C16:0	24.852±1.016	25.464±5.005	24.378±1.677	24.149±3.023
C18:0	9.290±0.397	10.657±1.204	9.477±1.962	10.047±1.921
C18:1n9c	23.034±1.403	25.772±1.152	24.362±2.554	25.760±4.189
C18:2n6t	0.225±0.019	0.257±0.101^c^	0.192±0.042^b^	0.202±0.031
C18:2n6c	3.343±0.235^b^	4.235±0.985^a^^c^	3.517±0.437^b^	3.390±0.472^b^
SCFA	1.704±0.046	2.232±0.422^c^	1.680±0.339^b^	1.798±0.385
DNS	28.999±5.289	32.193±8.154	30.192±3.165	28.546±4.739
MCFA	16.620±4.277	18.213±5.050	18.489±3.024	17.100±4.051
LCFA	80.441±3.906	77.380±6.342	78.687±2.918	79.911±3.839
VLCFA	0.579±0.416	0.507±0.075	0.390±0.201	0.362±0.203
SFA	68.210±2.296	71.796±12.242	66.944±2.908	65.479±4.628^b^
MUFA	26.214±1.788	21.089±14.518	27.799±2.559	29.197±4.367^b^
n3-UFA	0.737±0.599^b^^c^	0.371±0.061^a^	0.322±0.054^a^	0.367±0.117^a^
n6-UFA	4.181±0.104	5.076±1.104^c^	4.181±0.456^b^	4.128±0.596^b^
n9-UFA	24.814±1.630	19.062±15.651^c^	26.310±2.527^b^	27.803±4.157^b^
PUFA	4.880±0.437	5.461±1.109^c^	4.502±0.463^b^	4.493±0.593^b^

a p<0.05 compared with early lactation;

b p<0.05 compared with mid-lactation;

c p<0.05 compared with peak lactation.

SD, standard deviation; SCFA, short-chain fatty acid; DNS, *de novo* synthesis fatty acid; MCFA, medium-chain fatty acid; LCFA, long-chain fatty acid; VLCFA, very long-chain fatty acid; SFA, saturated fatty acid; MUFA, monounsaturated fatty acid; UFA, unsaturated fatty acid; PUFA, polyunsaturated fatty acid.

**Table 3:** Some fatty acid contents of Chinese Holstein milk at different lactation periods (% of total fatty acid, mean±SD)

Fatty acid	Early lactation	Mid-lactation	Peak lactation	Late lactation
C4:0	2.918±0.785^b^^c^	2.142±0.936^a^	2.087±0.806^a^	2.097±0.839^a^
C6:0	1.661±0.419	2.275±0.891	2.071±0.582	1.917±0.984
C8:0	0.983±0.264^b^^c^	1.675±0.602^a^	1.598±0.516^a^	1.531±0.582^a^
C10:0	2.361±1.305^b^^c^	3.811±1.174^a^	3.642±1.049^a^	3.267±1.282
C12:0	2.524±1.322^b^^c^	4.093±1.053^a^	3.915±1.356^a^	3.603±1.159^a^
C14:0	9.296±3.472^b^^c^	12.155±2.009^a^	12.521±2.193^a^	11.744±1.550^a^
C15:0	0.715±0.319^c^	0.948±0.247	1.063±0.444^a^	0.987±0.238^a^
C16:0	32.142±6.042^c^	32.711±3.663^c^	35.682±3.879^b^	33.836±3.131^c^
C16:1n7	1.285±0.265	1.195±0.388	1.332±0.695	1.485±0.460^b^
C18:0	15.461±4.532^b^^c^	11.205±2.610^a^	11.482±2.942^a^	11.191±2.693^a^
C18:1n9c	25.715±5.444^b^^c^	20.588±4.287^a^^c^	18.354±3.343^a^^b^	21.283±3.963^a^^c^
C18:2n6t	0.489±0.306	0.527±0.216	0.495±0.220	0.580±0.263
C18:2n6c	3.503±0.881	3.834±0.457	3.616±0.616	4.165±0.681^a^^c^
SCFA	2.918±0.785^b^^c^	2.142±0.936^a^	2.087±0.806^a^	2.097±0.839^a^
DNS	20.745±5.805^b^^c^	28.420±5.640^a^	28.128±5.135^a^	26.620±5.766^a^
MCFA	7.259±2.328^b^^c^	11.938±3.453^a^	11.084±2.825^a^	10.377±3.667^a^
LCFA	90.569±2.280^b^^c^	86.129±3.931^a^	87.229±3.533^a^	88.351±4.240^b^
VLCFA	0.454±0.297^c^	0.426±0.271^c^	0.219±0.213^a^^b^	0.293±0.363
SFA	68.708±7.977^c^	72.245±4.550^c^	74.931±3.747^a^^b^	71.382±4.323^c^
MUFA	28.012±6.034^b^^c^	23.415±4.201^a^^c^	21.136±3.520^a^^b^	24.642±4.118^a^^c^
n3-UFA	0.141±0.160	0.135±0.154	0.082±0.164	0.118±0.313
n6-UFA	4.338±1.051	4.840±0.567	4.471±0.794	4.977±0.863^a^^c^
n9-UFA	26.253±5.598^b^^c^	21.178±4.268^a^^c^	18.677±3.449^a^^b^	21.783±4.103^a^^c^
PUFA	4.479±1.140	4.975±0.593	4.553±0.876	5.095±0.902^c^

a p<0.05 compared with early lactation;

b p<0.05 compared with mid-lactation;

c p<0.05 compared with peak lactation.

SD, standard deviation; SCFA, short-chain fatty acid; DNS, *de novo* synthesis fatty acid ; MCFA, medium-chain fatty acid; LCFA, long-chain fatty acid; SFA, saturated fatty acid; MUFA, monounsaturated fatty acid; UFA, unsaturated fatty acid; PUFA, polyunsaturated fatty acid.

**Table 4:** Some fatty acid contents of Jersey dairy cattle milk at different lactation periods (% of total fatty acid, mean±SD)

Fatty acid	Early lactation	Mid-lactation	Peak lactation	Late lactation
C4:0	2.539±0.420^b^^c^	2.093±0.398^a^^c^	0.902±0.946^a^^b^	0.440±0.731^a^^b^^c^
C6:0	1.732±0.331^c^	1.572±0.255^c^	1.972±0.469^a^^b^	2.126±0.322^a^^b^
C8:0	0.962±0.186^c^	0.949±0.189^c^	1.375±0.428^a^^b^	1.520±0.300^a^^b^
C10:0	1.947±0.486^c^	2.167±0.575^c^	3.523±1.237^a^^b^	3.993±0.853^a^^b^^c^
C12:0	2.338±0.577^c^	2.802±0.797^c^	4.561±1.436^a^^b^	5.065±0.956^a^^b^
C14:0	7.894±0.917^b^^c^	8.833±1.877^a^^c^	11.600±1.820^a^^b^	12.102±1.100^a^^b^
C15:0	0.570±0.154^b^^c^	0.803±0.260^a^^c^	1.177±0.219^a^^b^	1.139±0.248^a^^b^
C16:0	31.943±2.756^b^^c^	34.688±4.810^a^^c^	38.436±3.150^a^^b^	36.555±2.627^a^^b^^c^
C16:1n7	1.633±0.371	1.500±0.447	1.431±0.396	1.517±1.080
C18:0	13.291±1.009^b^^c^	11.860±2.443^a^^c^	9.177±1.939^a^^b^	9.263±1.116^a^^b^
C18:1n9c	24.715±3.717^b^^c^	21.453±6.315^a^^c^	14.756±4.563^a^^b^	15.748±3.473^a^^b^
C18:2n6t	0.690±0.186	0.749±0.569^c^	0.508±0.149^b^	0.596±0.514
C18:2n6c	2.517±0.689^b^^c^	2.926±0.356^a^	2.779±0.377^a^	2.546±0.577^b^
SCFA	2.539±0.420^b^^c^	2.093±0.398^a^^c^	0.902±0.946^a^^b^	0.440±0.731^a^^b^^c^
DNS	19.088±2.441^c^	20.438±3.429^c^	26.680±4.634^b^	28.024±2.975^a^^b^
MCFA	7.076±1.443^c^	7.597±1.700^c^	11.594±3.500^a^^b^	12.849±2.377^a^^b^
LCFA	88.896±1.688^c^	88.728±1.915^c^	85.115±6.318^a^^b^	85.296±1.970^a^^b^
VLCFA	1.489±0.597	1.582±0.937^c^	1.205±0.513^b^	1.415±0.482
SFA	65.395±4.046^b^^c^	67.812±5.874^a^^c^	73.831±3.883^a^^b^	73.816±2.511^a^^b^
MUFA	29.170±4.093^b^^c^	26.346±5.764^a^^c^	20.069±4.925^a^^b^	21.142±2.651^a^^b^
n3-UFA	1.017±0.398^c^	0.886±0.386^c^	0.562±0.330^a^^b^	0.623±0.281^a^^b^
n6-UFA	4.237±0.767^b^	4.770±0.850^a^^c^	4.242±0.859^b^	4.299±0.472^b^
n9-UFA	26.243±3.821^b^^c^	23.437±5.422^a^^c^	17.142±4.625^a^^b^	17.975±2.195^a^^b^
PUFA	5.435±0.949	5.842±1.099^c^	4.915±1.105^b^	5.042±0.675^b^

a p<0.05 compared with early lactation;

b p<0.05 compared with mid-lactation;

c p<0.05 compared with peak lactation.

SD, standard deviation; SCFA, short-chain fatty acid;DNS, *de novo* synthesis fatty acid ; MCFA, medium-chain fatty acid; LCFA, long-chain fatty acid; VLCFA, very long-chain fatty acid; SFA, saturated fatty acid; MUFA, monounsaturated fatty acid; UFA, unsaturated fatty acid; PUFA, polyunsaturated fatty acid.

**Table 5:** Compositional analysis of the different types of milk fatty acids (% of total fatty acid, mean±SD)

Fatty acid	Breast milk	Saanen goat’s milk	Chinese Holstein cow’s Milk	Jersey cow’s Milk
C4:0	ND	1.786±0.374	2.164±0.861	1.398±1.071
C6:0	ND	2.166±0.399	2.028±0.836	1.863±0.419
C8:0	0.155±0.047^b^^c^	2.612±0.535^a^^c^	1.547±0.565^a^^b^	1.225±0.394^a^^b^^c^
C10:0	1.167±0.344^b^^c^	8.565±1.948^a^^c^	3.436±1.230^a^^b^	2.999±1.221^a^^b^^c^
C11:0	ND	0.298±0.106	0.561±0.821	0.133±0.112
C12:0	3.040±1.000^b^^c^	4.005±1.060^a^	3.725±1.265^a^	3.823±1.521^a^
C13:0	0.021±0.010^b^^c^	0.212±0.183^a^^c^	0.387±0.656^a^^b^	0.212±0.149^a^^c^
C14:0	4.960±0.945^b^^c^	8.580±1.141^a^^c^	11.905±2.176^a^^b^	10.333±2.306^a^^b^^c^
C14:1n5	0.133±0.135^c^	0.173±0.087^c^	0.949±0.638^a^^b^	0.843±0.282^a^^b^^c^
C15:0	0.177±0.039^b^^c^	1.091±0.206^a^^c^	0.986±0.332^a^^b^	0.962±0.329^a^^b^
C15:1n5	ND	ND	0.299±0.250	0.248±0.082
C16:0	24.451±2.208^c^	24.408±2.612^c^	34.096±3.906^a^^b^	35.852±4.166^a^^b^^c^
C16:1n7	1.861±0.314^b^^c^	0.878±0.284^a^^c^	1.361±0.534^a^^b^	1.506±0.625^a^^b^^c^
C17:0	0.256±0.036^b^^c^	2.394±1.495^a^^c^	0.682±0.343^a^^b^	0.503±0.085^a^^b^^c^
C17:1n7	0.101±0.025^b^^c^	0.433±0.153^a^^c^	0.290±0.148^a^^b^	0.232±0.147^a^^b^^c^
C18:0	5.557±0.892^b^^c^	9.833±1.793^a^^c^	11.607±3.083^a^^b^	10.636±2.450^a^^c^
C18:1n9c	31.099±1.858^b^^c^	25.004±3.387^a^^c^	20.494±4.364^a^^b^	18.517±6.135^a^^b^^c^
C18:2n6t	ND	0.206±0.045^a^^c^	0.532±0.241	0.622±0.400^a^^c^
C18:2n6c	20.389±2.900^b^^c^	3.505±0.533	3.866±0.678^a^^b^	2.713±0.514
C18:3n6	1.862±0.305^b^^c^	0.065±0.044^a^^c^	0.252±0.363^a^^b^	0.344±0.107^a^^b^
C18:3n3	0.199±0.052^b^^c^	0.101±0.021^a^^c^	0.446±0.164^a^^b^	0.177±0.129^b^^c^
C20:0	0.166±0.023^b^	0.655±0.407^a^^c^	0.237±0.152^b^	0.251±0.362^a^^b^
C20:1	0.364±0.230^b^^c^	0.466±0.076^a^^c^	0.248±0.153^a^^b^	0.194±0.101^a^^b^
C20:2	0.440±0.111^b^^c^	0.048±0.047^a^^c^	0.226±0.135^a^^b^	0.147±0.101^a^^b^^c^
C20:3n6	0.315±0.113^b^^c^	0.090±0.063^a^^c^	0.204±0.143^a^^b^	0.245±0.154^a^^b^
C20:3n3	0.058±0.034^b^^c^	0.111±0.036^a^	0.127±0.091^a^	0.178±0.122^a^^b^^c^
C20:4n6	0.106±0.073^b^	0.322±0.085^a^^c^	0.093±0.095^b^	0.181±0.125^a^^b^^c^
C20:5n3	0.319±0.341^b^^c^	0.095±0.042^a^	0.050±0.036^a^	0.184±0.138^a^^b^
C21:0	0.051±0.013^c^	0.059±0.040^c^	0.113±0.095^a^^b^	0.165±0.108^a^^b^
C22:0	0.110±0.040^c^	0.104±0.055^c^	0.254±0.193^a^^b^	0.183±0.128^a^^b^^c^
C22:1n9	0.151±0.055^b^^c^	0.041±0.091^a^^c^	0.301±0.139^a^^b^	0.147±0.092^b^^c^
C22:2n6	0.146±0.138	0.030±0.030	ND	0.329±0.228
C22:6n3	0.469±0.164^b^^c^	0.082±0.158^a^	0.213±0.068^a^	0.211±0.144^a^^b^
C23:0	0.362±0.346	0.040±0.023	ND	0.192±0.137
C24:0	0.118±0.078	ND	ND	0.181±0.282
C24:1n9	0.295±0.455	0.175±0.158	0.107±0.070	0.182±0.121^a^
SCFA	ND	1.786±0.374	2.164±0.861	1.398±1.071
DNS	9.138±1.716^b^^c^	29.489±4.535^a^^c^	27.054±5.800^a^^b^	24.015±5.183^a^^b^^c^
MCFA	3.858±1.576^b^^c^	17.646±3.700^a^^c^	10.700±3.428^a^^b^	10.044±3.487^a^^b^^c^
LCFA	91.813±3.916^b^^c^	79.303±3.697^a^^c^	87.689±3.964^a^^b^	86.763±4.312^a^^b^
VLCFA	1.548±0.647^b^^c^	0.404±0.219^a^	0.307±0.305^a^	1.400±0.661^a^^b^^c^
SFA	40.011±2.571^b^^c^	66.795±5.026^a^^c^	72.543±4.853^a^^b^	70.750±5.533^a^^b^^c^
MUFA	33.892±1.846^b^^c^	27.725±5.393^a^^c^	23.473±4.507^a^^b^	23.583±5.826^a^^b^
n3-UFA	2.441±0.678^b^^c^	0.384±0.205^a^^c^	0.111±0.231^a^^b^	0.742±0.390^a^^b^^c^
n6-UFA	20.701±3.967^b^^c^	4.235±0.614^a^	4.733±0.829^a^	4.385±0.789^a^
n9-UFA	31.899±1.809^b^^c^	26.241±5.572^a^^c^	20.956±4.482^a^^b^	20.606±5.613^a^^b^
PUFA	23.316±4.025^b^^c^	4.617±0.634^a^	4.844±0.885^a^	5.272±1.048^a^

a p<0.05 compared with breast milk;

b p<0.05 compared with Saanen goat’s milk;

c p<0.05 compared with Chinese Holstein cow’s milk.

SD, standard deviation; ND, not detected; SCFA, short-chain fatty acid; DNS, *de novo* synthesis fatty acid ; MCFA, medium-chain fatty acid; LCFA, long-chain fatty acid; VLCFA, very long-chain fatty acid; SFA, saturated fatty acid; MUFA, monounsaturated fatty acid; UFA, unsaturated fatty acid; PUFA, polyunsaturated fatty acid.

## SUPPLEMENTARY MATERIAL

**Supplement 1**. GC-FID analysis of a FAME standard fatty acid mixture on a 60 m x 0.25 mm ID, 0.15 μm DB-23 column.

**Supplement 2**. GC-FID analysis of a FAME standard fatty acid mixture on a 60 m x 0.25 mm ID, 0.25 μm DB-23 column.

**Supplement 3**. GC-FID analysis of a Jersey bovine milk fatty acid sample on a 60 m x 0.25 mm ID, 0.25 μm DB-23 column.

**Supplement 4**. The fatty acid contents of breast milk at different lactation (% of total fatty acid, Mean±SD).

**Supplement 5**. The fatty acid contents of goat milk at different lactation (% of total fatty acid, Mean±SD).

**Supplement 6**. The fatty acid contents of Chinese Holstein milk at different lactation (% of total fatty acid, Mean±SD).

**Supplement 7**. The fatty acid contents of Jersey dairy cattle milk at different lactation (% of total fatty acid, Mean±SD).
