# Supplementary material for: Differential analysis of milk fatty acids in human, Saanen goat, Holstein cow, and Jersey cow milk at different stages of lactation
Source: Anim Biosci. 2025 Mar 31;38(10):2233–49. doi: 10.5713/ab.24.0528 (PMC12415369; doi:10.5713/ab.24.0528)
Supplement: Supplementary file 1 [file ab-24-0528-Supplementary-1,2.pdf]

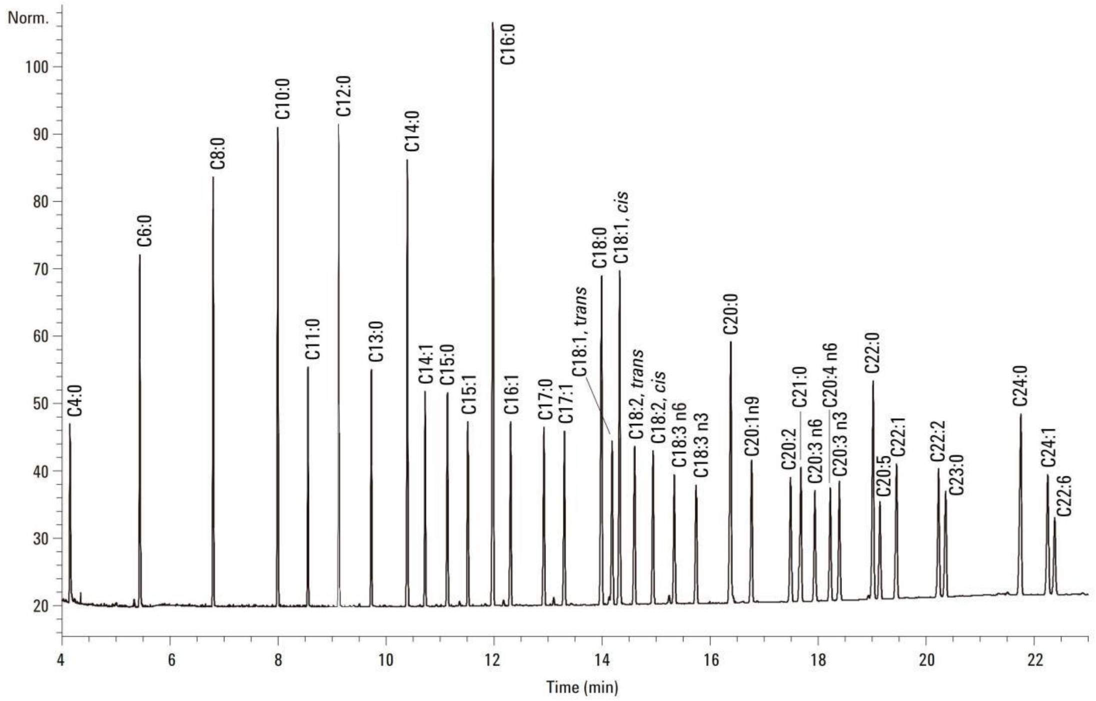

Supplement 1. GC-FID analysis of a FAME standard fatty acid mixture on a 60 m x 0.25 mm ID, 0.15 µm DB-23 column.

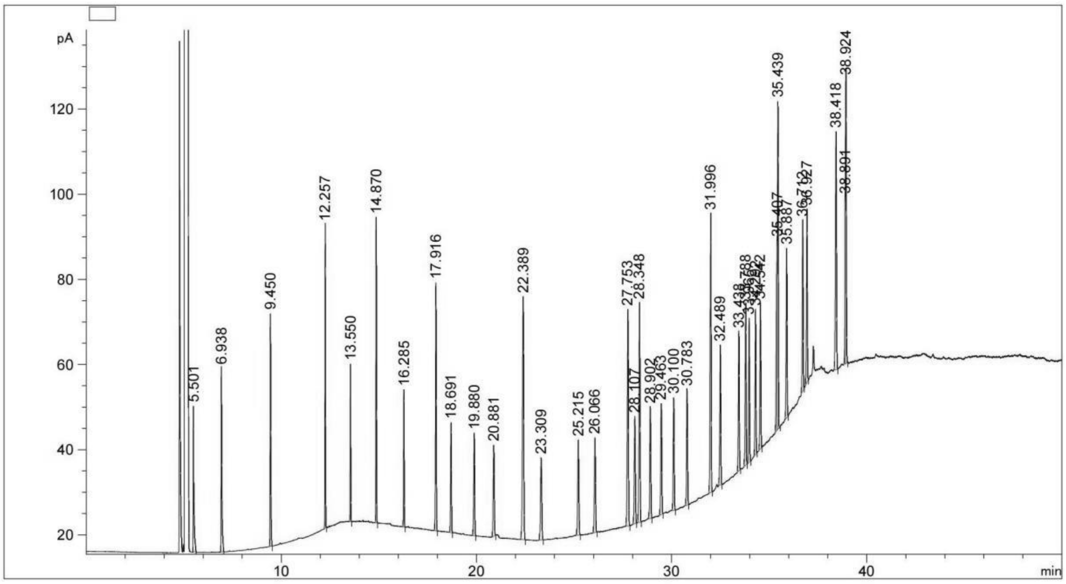

Supplement 2. GC-FID analysis of a FAME standard fatty acid mixture on a 60 m x 0.25 mm ID, 0.25 µm DB-23 column.
