# Supplementary figures and images for: Differential analysis of milk fatty acids in human, Saanen goat, Holstein cow, and Jersey cow milk at different stages of lactation
Source: Anim Biosci. 2025 Mar 31;38(10):2233–49. doi: 10.5713/ab.24.0528 (PMC12415369; doi:10.5713/ab.24.0528)

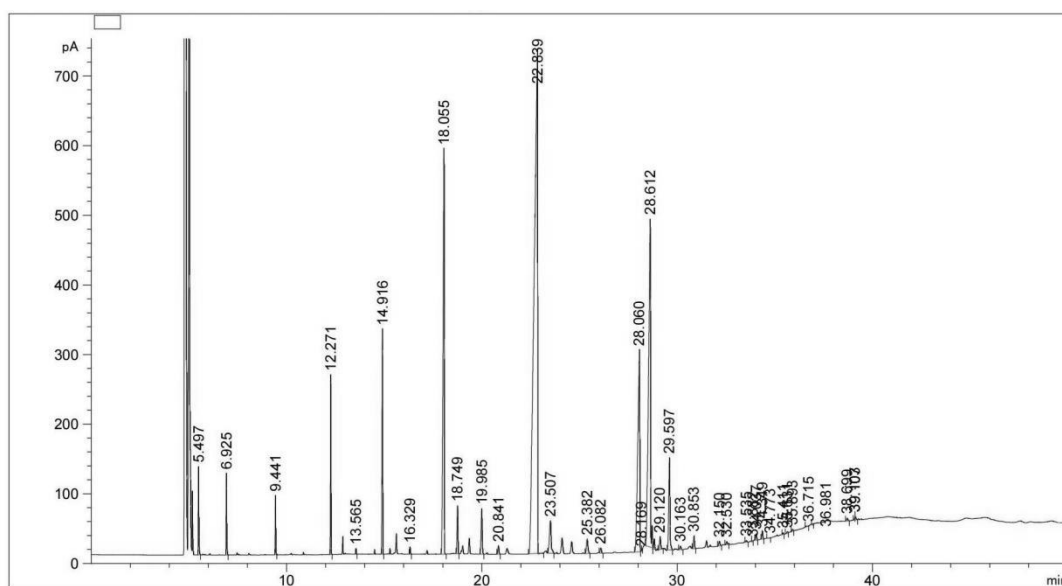

**Supplement 3.** GC-FID analysis of a Jersey bovine milk fatty acid sample on a 60 m x 0.25 mm ID, 0.25  $\mu$ m DB-23 column.

Supplement: Supplementary file 2 [file ab-24-0528-Supplementary-3.pdf]
