# Supplementary material for: Differential analysis of milk fatty acids in human, Saanen goat, Holstein cow, and Jersey cow milk at different stages of lactation
Source: Anim Biosci. 2025 Mar 31;38(10):2233–49. doi: 10.5713/ab.24.0528 (PMC12415369; doi:10.5713/ab.24.0528)
Supplement: Supplementary file 3 [file ab-24-0528-Supplementary-4.pdf]

Supplement 4. The fatty acid contents of breast milk at different lactation (% of total fatty acid，Mean±SD)

Fatty acid	Colostrum stage	Transitional milk stage	Mature milk stage
C8:0	0.115±0.012[b]	0.131±0.011[a]	0.206±0.041[a,b]
C10:0	0.677±0.385[b]	1.261±0.261[a]	1.249±0.233[a,b]
C12:0	1.431±0.256[b]	3.140±0.586[a]	3.640±0.869[a,b]
C13:0	0.017±0.005[b]	0.024±0.013[a]	0.020±0.004
C14:0	5.018±0.613	5.219±0.912	4.585±1.000[b]
C14:1n5	0.065±0.027	0.125±0.185	0.173±0.031[a]
C15:0	0.137±0.028[b]	0.172±0.038[a]	0.200±0.028[a,b]
C16:0	27.426±1.637[b]	23.755±1.475[a]	24.104±2.237[a]
C16:1n7	1.463±0.249[b]	1.856±0.254[a]	2.039±0.250[a,b]
C17:0	0.200±0.021[b]	0.267±0.025[a]	0.263±0.033[a]
C17:1n7	0.062±0.016[b]	0.109±0.018[a]	0.107±0.018[a]
C18:0	5.366±0.641	5.337±0.906	5.938±0.857[a,b]
C18:1n9c	30.098±1.827[b]	31.664±1.915[a]	30.768±1.548[b]
C18:2n6(LA)	19.452±5.176	20.746±2.175	20.312±2.378
C18:3n3	1.659±0.100[b]	2.139±0.074[a]	1.539±0.140[a,b]
C18:3n6	0.158±0.025[b]	0.225±0.013[a]	0.182±0.074[b]
C20:0	0.146±0.020[b]	0.173±0.022[a]	0.167±0.021[a]
C20:1n9	0.336±0.092	0.383±0.326	0.352±0.062
C20:2n6	0.567±0.052[b]	0.439±0.124[a]	0.387±0.060[a]
C20:3n6	0.293±0.089	0.311±0.109	0.330±0.129
C20:3n3	0.100±0.035[b]	0.042±0.024[a]	0.061±0.030[a,b]
C20:4n6	0.240±0.060[b]	0.103±0.038[a]	0.051±0.026[a,b]
C20:5n3	0.894±0.340[b]	0.289±0.180[a]	0.088±0.136[a,b]
C21:0	0.045±0.013	0.052±0.014	0.054±0.012[a]

C22:0	0.098±0.026	0.113±0.053	0.110±0.018
C22:1n9	0.186±0.080[b]	0.113±0.030[a]	0.187±0.023[b]
C22:2n6	0.222±0.060[b]	0.117±0.119[a]	0.151±0.172
C22:6n3	0.603±0.205[b]	0.531±0.101[a]	0.322±0.093[a,b]
C23:0	0.232±0.153	0.219±0.113	0.611±0.461[a,b]
C24:0	0.227±0.094[b]	0.125±0.048[a]	0.059±0.042[a,b]
C24:1n9	0.541±1.031	0.314±0.259	0.162±0.058[a]
DNS	7.296±0.754[b]	9.292±1.611[a]	9.726±1.636[a]
MCFA	2.061±0.570[b]	3.774±1.511[a]	4.749±1.238[a,b]
LCFA	92.841±5.976	92.021±4.119	91.088±2.097
VLCFA	2.075±1.052[b]	1.366±0.487[a]	1.564±0.484[a]
SFA	40.927±1.871[b]	39.124±2.578[a]	40.813±2.470[b]
MUFA	32.681±1.218[b]	34.478±1.953[a]	33.624±1.625[b]
n3-UFA	2.802±0.683	2.760±0.489	1.854±0.477[a,b]
n6-UFA	20.419±5.103	20.602±4.542	20.958±2.356
n9-UFA	31.183±1.381[b]	32.443±1.971[a]	31.474±1.539[b]
PUFA	23.368±4.907	23.560±4.652	22.964±2.491

Note: a: $p < 0.05$ compared with Colostrum stage; b: $p < 0.05$ compared with Transitional milk stage. DNS(de novo synthesis fatty acid), MCFA(Medium-chain fatty acid), LCFA(Long-chain fatty acid), VLCFA(Very long-chain fatty acid), SFA(saturated fatty acid), MUFA(monounsaturated fatty acid), PUFA(polyunsaturated fatty acid).
