# Supplementary material for: Differential analysis of milk fatty acids in human, Saanen goat, Holstein cow, and Jersey cow milk at different stages of lactation
Source: Anim Biosci. 2025 Mar 31;38(10):2233–49. doi: 10.5713/ab.24.0528 (PMC12415369; doi:10.5713/ab.24.0528)
Supplement: Supplementary file 4 [file ab-24-0528-Supplementary-5.pdf]

Supplement 5. The fatty acid contents of goat milk at different lactation (% of total fatty acid，Mean±SD)

Fatty acid	Early lactation	Mid-lactation	Peak lactation	Late lactation
C4:0	1.704±0.046	2.232±0.422^{c}	1.680±0.339^{b}	1.798±0.385
C6:0	2.013±0.251^{b}	2.759±0.812^{a,c}	2.215±0.278^{b}	2.046±0.323^{b}
C8:0	2.496±0.621	2.672±0.750	2.751±0.473	2.518±0.557
C10:0	7.660±2.106	8.395±2.547	9.085±1.622	8.377±2.130
C11:0	0.294±0.120	0.314±0.195	0.330±0.121	0.272±0.074
C12:0	4.158±1.731	4.073±1.666	4.107±0.808	3.886±1.094
C13:0	0.166±0.042	0.221±0.125	0.278±0.293	0.170±0.049
C14:0	9.280±0.861	9.826±2.424	8.503±0.431	8.273±1.153^{b}
C14:1n5	0.164±0.006	0.218±0.173	0.170±0.074	0.169±0.092
C15:0	1.065±0.108^{b}	1.482±0.509^{a,c}	1.073±0.082^{b}	1.037±0.134^{b}
C16:0	24.852±1.016	25.464±5.005	24.378±1.677	24.149±3.023
C16:1n7	0.786±0.168	1.148±0.672	0.926±0.275	0.808±0.186
C17:0	3.906±3.397	2.659±1.355	2.278±1.256	2.149±1.195
C17:1n7	0.451±0.142	0.661±0.290^{c}	0.393±0.083^{b}	0.417±0.144^{b}
C18:0	9.290±0.397	10.657±1.204	9.477±1.962	10.047±1.921
C18:1n9c	23.034±1.403	25.772±1.152	24.362±2.554	25.760±4.189
C18:2n6t	0.225±0.019	0.257±0.101^{c}	0.192±0.042^{b}	0.202±0.031
C18:2n6c	3.343±0.235^{b}	4.235±0.985^{a,c}	3.517±0.437^{b}	3.390±0.472^{b}
C18:3n6	0.131±0.085^{b,c}	0.052±0.028^{a}	0.062±0.026^{a}	0.056±0.040^{a}
C18:3n3	0.131±0.045^{c}	0.108±0.024	0.096±0.008^{a}	0.099±0.019^{a}
C20:0	1.098±0.953	0.713±0.298	0.615±0.346	0.592±0.314
C20:1n9	0.456±0.032^{b}	0.588±0.119^{a,c}	0.448±0.048^{b}	0.459±0.076^{b}
C20:2n6	0.029±0.006	0.046±0.019	0.048±0.053	0.051±0.051
C20:3n6	0.116±0.062	0.089±0.036	0.080±0.060	0.092±0.072
C20:3n3	0.151±0.072	0.098±0.019	0.107±0.022	0.110±0.036
C20:4n6	0.347±0.084	0.394±0.076	0.293±0.101	0.326±0.071
C20:5n3	0.120±0.021	0.103±0.025	0.074±0.016	0.104±0.054
C21:0	0.092±0.047	0.060±0.022	0.061±0.050	0.050±0.030
C22:0	0.124±0.051^{b}	0.203±0.141^{a,c}	0.092±0.025^{b}	0.091±0.029^{b}
C22:1n9	0.020±0.008	ND	0.080±0.148	0.020±0.010
C22:2n6	ND	0.027±0.016	0.025±0.021	0.034±0.037
C22:6n3	0.336±0.501^{b,c}	0.062±0.017^{a}	0.049±0.025^{a}	0.058±0.057^{a}
C23:0	ND	0.066±0.019	0.034±0.022	0.038±0.022
C24:1n9	0.137±0.010	0.153±0.114	0.207±0.193	0.162±0.154
SCFA	1.704±0.046	2.232±0.422^{c}	1.680±0.339^{b}	1.798±0.385
DNS	28.999±5.289	32.193±8.154	30.192±3.165	28.546±4.739
MCFA	16.620±4.277	18.213±5.050	18.489±3.024	17.100±4.051
LCFA	80.441±3.906	77.380±6.342	78.687±2.918	79.911±3.839
VLCFA	0.579±0.416	0.507±0.075	0.390±0.201	0.362±0.203
SFA	68.210±2.296	71.796±12.242	66.944±2.908	65.479±4.628^{b}
MUFA	26.214±1.788	21.089±14.518	27.799±2.559	29.197±4.367^{b}
n3-UFA	0.737±0.599^{b,c}	0.371±0.061^{a}	0.322±0.054^{a}	0.367±0.117^{a}
n6-UFA	4.181±0.104	5.076±1.104^{c}	4.181±0.456^{b}	4.128±0.596^{b}
n9-UFA	24.814±1.630	19.062±15.651^{c}	26.310±2.527^{b}	27.803±4.157^{b}
PUFA	4.880±0.437	5.461±1.109^{c}	4.502±0.463^{b}	4.493±0.593^{b}

Note: a: p<0.05 compared with early lactation; b: p<0.05 compared with mid-lactation; c: p<0.05 compared with peak lactation.DNS(de novo synthesis fatty acid), MCFA(Medium-chain fatty acid), LCFA(Long-chain fatty acid), VLCFA(Very long-chain fatty acid), SFA(saturated fatty acid), MUFA(monounsaturated fatty acid), PUFA(polyunsaturated fatty acid).
