# Supplementary material for: Differential analysis of milk fatty acids in human, Saanen goat, Holstein cow, and Jersey cow milk at different stages of lactation
Source: Anim Biosci. 2025 Mar 31;38(10):2233–49. doi: 10.5713/ab.24.0528 (PMC12415369; doi:10.5713/ab.24.0528)
Supplement: Supplementary file 5 [file ab-24-0528-Supplementary-6.pdf]

Supplement 6. The fatty acid contents of Chinese Holstein milk at different lactation (% of total fatty acid，Mean±SD)

Fatty acid	Early lactation	Mid-lactation	Peak lactation	Late lactation
C4:0	2.918±0.785[b,c]	2.142±0.936[a]	2.087±0.806[a]	2.097±0.839[a]
C6:0	1.661±0.419	2.275±0.891	2.071±0.582	1.917±0.984
C8:0	0.983±0.264[b,c]	1.675±0.602[a]	1.598±0.516[a]	1.531±0.582[a]
C10:0	2.361±1.305[b,c]	3.811±1.174[a]	3.642±1.049[a]	3.267±1.282
C11:0	0.120±0.037	0.144±0.097[c]	0.887±1.251[b]	0.561±0.416
C12:0	2.524±1.322[b,c]	4.093±1.053[a]	3.915±1.356[a]	3.603±1.159[a]
C13:0	0.201±0.226	0.403±0.554	0.522±1.066	0.314±0.250
C14:0	9.296±3.472[b,c]	12.155±2.009[a]	12.521±2.193[a]	11.744±1.550[a]
C14:1n5	0.497±0.249[c]	0.829±0.350	1.049±0.691[a]	1.006±0.721
C15:0	0.715±0.319[c]	0.948±0.247	1.063±0.444[a]	0.987±0.238[a]
C15:1	0.248±0.023	0.172±0.152	0.320±0.274	0.352±0.273
C16:0	32.142±6.042[c]	32.711±3.663[c]	35.682±3.879[b]	33.836±3.131[c]
C16:1n7	1.285±0.265	1.195±0.388	1.332±0.695	1.485±0.460[b]
C17:0	0.638±0.078	0.594±0.384	0.585±0.267	0.815±0.374[b,c]
C17:1n7	0.276±0.077	0.228±0.092	0.198±0.100	0.384±0.159[b,c]
C18:0	15.461±4.532[b,c]	11.205±2.610[a]	11.482±2.942[a]	11.191±2.693[a]
C18:1n9c	25.715±5.444[b,c]	20.588±4.287[a,c]	18.354±3.343[a,b]	21.283±3.963[a,c]
C18:2n6t	0.489±0.306	0.527±0.216	0.495±0.220	0.580±0.263
C18:2n6c	3.503±0.881	3.834±0.457	3.616±0.616	4.165±0.681[a,c]
C18:3n3	0.197±0.106	0.135±0.153	0.231±0.226	0.495±0.704
C18:3n6	0.370±0.064	0.486±0.138	0.441±0.124	0.442±0.216
C20:0	0.182±0.070	0.262±0.243	0.216±0.093	0.271±0.131
C20:1n9	0.158±0.053	0.138±0.054[c]	0.278±0.184[b]	0.325±0.149[a,b]
C20:2n6	ND	0.212±0.232	0.298±0.110	0.167±0.120
C20:3n6	0.190±0.054	0.311±0.144	0.169±0.126	0.086±0.093[b]
C20:3n3	ND	0.099±0.119	0.085±0.080	0.170±0.058
C20:4n6	0.048±0.026	0.062±0.024	0.089±0.105	0.180±0.155
C20:5n3	ND	0.059±0.035	ND	ND
C21:0	0.101±0.040	0.109±0.137	0.089±0.080	0.149±0.085
C22:0	0.131±0.053	0.269±0.211	0.240±0.118	0.311±0.269
C22:1n9	0.247±0.035	0.322±0.164	0.297±0.139	0.304±0.143
C22:6n3	0.226±0.044	0.242±0.051	ND	ND
C24:1n9	0.133±0.082	0.064±0.050	ND	0.125±0.062
SCFA	2.918±0.785[b,c]	2.142±0.936[a]	2.087±0.806[a]	2.097±0.839[a]
DNS	20.745±5.805[b,c]	28.420±5.640[a]	28.128±5.135[a]	26.620±5.766[a]
MCFA	7.259±2.328[b,c]	11.938±3.453[a]	11.084±2.825[a]	10.377±3.667[a]
LCFA	90.569±2.280[b,c]	86.129±3.931[a]	87.229±3.533[a]	88.351±4.240[b]
VLCFA	0.454±0.297[c]	0.426±0.271[c]	0.219±0.213[a,b]	0.293±0.363
SFA	68.708±7.977[c]	72.245±4.550[c]	74.931±3.747[a,b]	71.382±4.323[c]
MUFA	28.012±6.034[b,c]	23.415±4.201[a,c]	21.136±3.520[a,b]	24.642±4.118[a,c]
n3-UFA	0.141±0.160	0.135±0.154	0.082±0.164	0.118±0.313
n6-UFA	4.338±1.051	4.840±0.567	4.471±0.794	4.977±0.863[a,c]
n9-UFA	26.253±5.598[b,c]	21.178±4.268[a,c]	18.677±3.449[a,b]	21.783±4.103[a,c]
PUFA	4.479±1.140	4.975±0.593	4.553±0.876	5.095±0.902[c]

Note: a: $p<0.05$ compared with early lactation; b: $p<0.05$ compared with mid-lactation; c: $p<0.05$ compared with peak lactation. DNS(de novo synthesis fatty acid), MCFA(Medium-chain fatty acid), LCFA(Long-chain fatty acid), VLCFA(Very long-chain fatty acid), SFA(saturated fatty acid), MUFA(monounsaturated fatty acid), PUFA(polyunsaturated fatty acid).
