# Supplementary material for: Differential analysis of milk fatty acids in human, Saanen goat, Holstein cow, and Jersey cow milk at different stages of lactation
Source: Anim Biosci. 2025 Mar 31;38(10):2233–49. doi: 10.5713/ab.24.0528 (PMC12415369; doi:10.5713/ab.24.0528)
Supplement: Supplementary file 6 [file ab-24-0528-Supplementary-7.pdf]

Supplement 7. The fatty acid contents of Jersey dairy cattle milk at different lactation (% of total fatty acid，Mean±SD)

Fatty acid	Early lactation	Mid-lactation	Peak lactation	Late lactation
C4:0	2.539±0.420^{b,c}	2.093±0.398^{a,c}	0.902±0.946^{a,b}	0.440±0.731^{a,b,c}
C6:0	1.732±0.331^{c}	1.572±0.255^{c}	1.972±0.469^{a,b}	2.126±0.322^{a,b}
C8:0	0.962±0.186^{c}	0.949±0.189^{c}	1.375±0.428^{a,b}	1.520±0.300^{a,b}
C10:0	1.947±0.486^{c}	2.167±0.575^{c}	3.523±1.237^{a,b}	3.993±0.853^{a,b,c}
C11:0	0.097±0.062^{c}	0.107±0.063^{c}	0.163±0.171^{a,b}	0.146±0.049
C12:0	2.338±0.577^{c}	2.802±0.797^{c}	4.561±1.436^{a,b}	5.065±0.956^{a,b}
C13:0	0.214±0.193	0.201±0.175	0.202±0.114	0.235±0.128
C14:0	7.894±0.917^{b,c}	8.833±1.877^{a,c}	11.600±1.820^{a,b}	12.102±1.100^{a,b}
C14:1n5	0.539±0.096^{b,c}	0.679±0.135^{a,c}	1.013±0.245^{a,b}	1.022±0.234^{a,b,c}
C15:0	0.570±0.154^{b,c}	0.803±0.260^{a,c}	1.177±0.219^{a,b}	1.139±0.248^{a,b}
C15:1n5	0.257±0.147	0.231±0.063	0.262±0.059	0.237±0.052
C16:0	31.943±2.756^{b,c}	34.688±4.810^{a,c}	38.436±3.150^{a,b}	36.555±2.627^{a,b,c}
C16:1n7	1.633±0.371	1.500±0.447	1.431±0.396	1.517±1.080
C17:0	0.529±0.055	0.524±0.055	0.491±0.083	0.478±0.119^{a,b}
C17:1n7	0.279±0.072^{c}	0.257±0.128^{c}	0.179±0.075^{a,b}	0.242±0.244
C18:0	13.291±1.009^{b,c}	11.860±2.443^{a,c}	9.177±1.939^{a,b}	9.263±1.116^{a,b}
C18:1n9c	24.715±3.717^{b,c}	21.453±6.315^{a,c}	14.756±4.563^{a,b}	15.748±3.473^{a,b}
C18:2n6t	0.690±0.186	0.749±0.569^{c}	0.508±0.149^{b}	0.596±0.514
C18:2n6c	2.517±0.689^{b,c}	2.926±0.356^{a}	2.779±0.377^{a}	2.546±0.577^{b}
C18:3n6	0.306±0.051	0.344±0.062	0.362±0.136	0.350±0.127
C18:3n3	0.233±0.119^{c}	0.248±0.140^{c}	0.143±0.115^{a,b}	0.102±0.083^{a,b}
C20:0	0.485±0.756^{b,c}	0.245±0.139^{a}	0.161±0.085^{a}	0.197±0.169^{a}
C20:1	0.218±0.076	0.242±0.106^{c}	0.175±0.111^{b}	0.149±0.071^{a,b}
C20:2	0.181±0.096^{c}	0.185±0.120^{c}	0.117±0.091^{a,b}	0.120±0.074^{a,b}
C20:3n6	0.279±0.077	0.295±0.282^{c}	0.217±0.046^{b}	0.202±0.068^{b}
C20:3n3	0.244±0.113^{c}	0.220±0.150^{c}	0.143±0.107^{a,b}	0.128±0.074^{a,b}
C20:4n6	0.258±0.153^{c}	0.225±0.110^{c}	0.138±0.106^{a,b}	0.133±0.093^{a,b}
C20:5n3	0.275±0.185^{b,c}	0.201±0.118^{a,c}	0.129±0.102^{a,b}	0.172±0.122^{a}
C21:0	0.201±0.114	0.188±0.100	0.159±0.132	0.121±0.034^{a,b}
C22:0	0.155±0.066^{b}	0.227±0.235^{a}	0.176±0.045	0.170±0.069
C22:1n9	0.160±0.087	0.147±0.085	0.152±0.097	0.129±0.098
C22:2n6	0.187±0.131^{c}	0.231±0.138^{c}	0.383±0.278^{a,b}	0.471±0.179^{a,b}
C22:6n3	0.265±0.179^{c}	0.217±0.125	0.169±0.091^{a}	0.221±0.181
C23:0	0.247±0.157^{c}	0.242±0.146^{c}	0.136±0.104^{a,b}	0.177±0.124
C24:0	0.251±0.208	0.310±0.499^{c}	0.109±0.078^{b}	0.089±0.061^{a,b}
C24:1n9	0.225±0.120^{c}	0.208±0.145	0.156±0.101^{a}	0.156±0.112^{a}
SCFA	2.539±0.420^{b,c}	2.093±0.398^{a,c}	0.902±0.946^{a,b}	0.440±0.731^{a,b,c}
DNS	19.088±2.441^{c}	20.438±3.429^{c}	26.680±4.634^{b}	28.024±2.975^{a,b}
MCFA	7.076±1.443^{c}	7.597±1.700^{c}	11.594±3.500^{a,b}	12.849±2.377^{a,b}
LCFA	88.896±1.688^{c}	88.728±1.915^{c}	85.115±6.318^{a,b}	85.296±1.970^{a,b}
VLCFA	1.489±0.597	1.582±0.937^{c}	1.205±0.513^{b}	1.415±0.482
SFA	65.395±4.046^{b,c}	67.812±5.874^{a,c}	73.831±3.883^{a,b}	73.816±2.511^{a,b}
MUFA	29.170±4.093^{b,c}	26.346±5.764^{a,c}	20.069±4.925^{a,b}	21.142±2.651^{a,b}
n3-UFA	1.017±0.398^{c}	0.886±0.386^{c}	0.562±0.330^{a,b}	0.623±0.281^{a,b}
n6-UFA	4.237±0.767^{b}	4.770±0.850^{a,c}	4.242±0.859^{b}	4.299±0.472^{b}
n9-UFA	26.243±3.821^{b,c}	23.437±5.422^{a,c}	17.142±4.625^{a,b}	17.975±2.195^{a,b}
PUFA	5.435±0.949	5.842±1.099^{c}	4.915±1.105^{b}	5.042±0.675^{b}

Note: a: $p<0.05$ compared with early lactation; b: $p<0.05$ compared with mid-lactation; c: $p<0.05$ compared with peak lactation. DNS(de novo synthesis fatty acid), MCFA(Medium-chain fatty acid), LCFA(Long-chain fatty acid), VLCFA(Very long-chain fatty acid), SFA(saturated fatty acid), MUFA(monounsaturated fatty acid), PUFA(polyunsaturated fatty acid).
